# Lipid membrane remodeling and metabolic response during isobutanol and ethanol exposure in *Zymomonas mobilis*

**DOI:** 10.1186/s13068-023-02450-9

**Published:** 2024-01-28

**Authors:** Julio Rivera Vazquez, Edna Trujillo, Jonathan Williams, Fukang She, Fitsum Getahun, Melanie M. Callaghan, Joshua J. Coon, Daniel Amador-Noguez

**Affiliations:** 1 DOE Great Lakes Bioenergy Research Center, University of Wisconsin-Madison, Madison, WI 53726 USA; 2 Department of Bacteriology, University of Wisconsin-Madison, Madison, WI USA; 3 Genome Center of Wisconsin, Madison, WI USA; 4 Morgridge Institute for Research, Madison, WI USA; 5 National Center for Quantitative Biology of Complex Systems, University of Wisconsin-Madison, Madison, WI USA; 6 Department of Chemistry, University of Wisconsin-Madison, Madison, WI USA; 7 Department of Biomolecular Chemistry, University of Wisconsin-Madison, Madison, WI USA

**Keywords:** Biofuels, Lipidomics, Proteomics, Cyclopropane fatty acids, *Zymomonas mobilis*, *Clostridium acetobutylicum*

## Abstract

**Background:**

Recent engineering efforts have targeted the ethanologenic bacterium *Zymomonas mobilis* for isobutanol production. However, significant hurdles remain due this organism’s vulnerability to isobutanol toxicity, adversely affecting its growth and productivity. The limited understanding of the physiological impacts of isobutanol on *Z. mobilis* constrains our ability to overcome these production barriers.

**Results:**

We utilized a systems-level approach comprising LC–MS/MS-based lipidomics, metabolomics, and shotgun proteomics, to investigate how exposure to ethanol and isobutanol impact the lipid membrane composition and overall physiology of *Z. mobilis*. Our analysis revealed significant and distinct alterations in membrane phospholipid and fatty acid composition resulting from ethanol and isobutanol exposure. Notably, ethanol exposure increased membrane cyclopropane fatty acid content and expression of cyclopropane fatty acid (CFA) synthase. Surprisingly, isobutanol decreased cyclopropane fatty acid content despite robust upregulation of CFA synthase. Overexpression of the native *Z. mobilis’* CFA synthase increased cyclopropane fatty acid content in all phospholipid classes and was associated with a significant improvement in growth rates in the presence of added ethanol and isobutanol. Heterologous expression of CFA synthase from *Clostridium acetobutylicum* resulted in a near complete replacement of unsaturated fatty acids with cyclopropane fatty acids, affecting all lipid classes. However, this did not translate to improved growth rates under isobutanol exposure.

Correlating with its greater susceptibility to isobutanol, *Z. mobilis* exhibited more pronounced alterations in its proteome, metabolome, and overall cell morphology—including cell swelling and formation of intracellular protein aggregates —when exposed to isobutanol compared to ethanol. Isobutanol triggered a broad stress response marked by the upregulation of heat shock proteins, efflux transporters, DNA repair systems, and the downregulation of cell motility proteins. Isobutanol also elicited widespread dysregulation of *Z. mobilis*’ primary metabolism evidenced by increased levels of nucleotide degradation intermediates and the depletion of biosynthetic and glycolytic intermediates.

**Conclusions:**

This study provides a comprehensive, systems-level evaluation of the impact of ethanol and isobutanol exposure on the lipid membrane composition and overall physiology of *Z. mobilis*. These findings will guide engineering of *Z. mobilis* towards the creation of isobutanol-tolerant strains that can serve as robust platforms for the industrial production of isobutanol from lignocellulosic sugars.

**Supplementary Information:**

The online version contains supplementary material available at 10.1186/s13068-023-02450-9.

## Introduction

The production of fuels and chemicals from plant biomass by microbes presents a sustainable alternative to fossil resources [1, 2]. However, for this process to be economically viable, high yields and titers are required [3–5]. *Zymomonas mobilis*, an aerotolerant anaerobic α-proteobacterium, is an attractive candidate for industrial-scale biofuel production [6] due to its high catabolic rate [7], low biomass generation, resistance against inhibitors present in lignocellulosic hydrolysates [8], and an increasing set of genetic engineering tools [9, 10]. *Z. mobilis* utilizes the highly thermodynamically favorable Entner-Doudoroff (ED) glycolytic pathway to convert up to 95% of consumed glucose to ethanol [6, 11–15]. While this trait makes it a promising chassis microbe for bioethanol production, its highly catabolic metabolism could be leveraged to produce other valuable bioproducts by rerouting carbon from ethanol production [16–18].

**Fig. 1:**
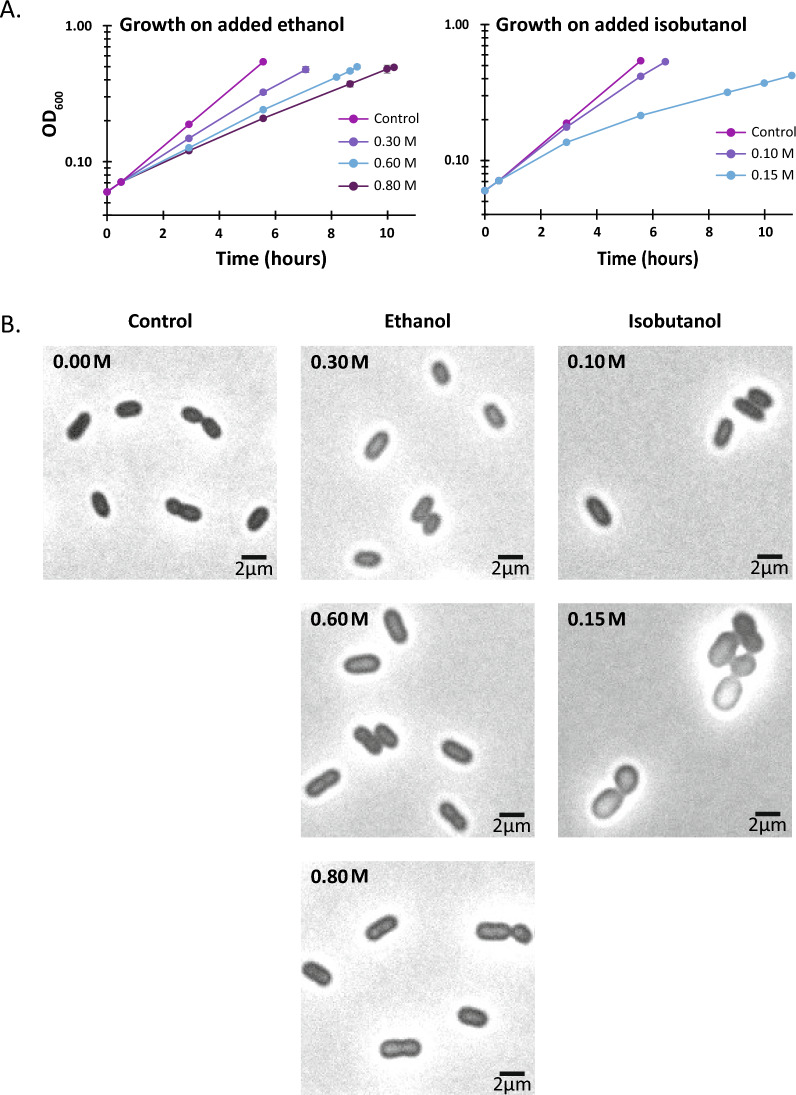
**A** Anaerobic growth of *Z. mobilis* ZM4 in minimal media containing varying amounts of added ethanol (0.3, 0.6, or 0.8 M) or isobutanol (0.1 and 0.15 M). *Z. mobili*s was more susceptible to growth inhibition by isobutanol than ethanol. Each data point represents the average of four biological replicates, with error bars indicating the standard deviation. Refer to Table S2 for doubling times and methods section for cell density measurements. **B** Bright-field microscopy evaluation of gross morphological alterations when *Z mobilis* was grown in minimal media supplemented with either ethanol (0.3, 0.6, or 0.8 M) or isobutanol (0.10 or 0.15 M)

Recent efforts have focused on engineering *Z. mobilis* to produce isobutanol, but many challenges remain [19, 20]. Despite its natural tolerance to high concentrations of ethanol, *Z. mobilis* is highly susceptible to growth inhibition by isobutanol, even at relatively low concentrations (Fig. 1). Recent studies indicate that isobutanol toxicity represents a significant barrier in realizing high-yield and high-titer production of this biofuel in engineered isobutanologenic *Z. mobilis* strains [20, 21]. For instance, a recent study underscored the need of using a nitrogen gas-stripping technique to continuously remove isobutanol from the culture medium to achieve high isobutanol yields in *Z. mobilis* [19]. Gas stripping has also proven beneficial for boosting isobutanol production in *Escherichia coli* as well [19, 22].

Several studies have investigated *Z. mobilis*’ response to stressors such as ethanol, organic acids, and oxygen [23–25]. Transcriptomic and proteomic analysis have shown that ethanol stress affects numerous cellular processes [26, 27], including upregulation of chaperones involved in protein folding and downregulation of cell motility proteins [27, 28]. A previous study examining the effects of ethanol on the lipid composition of *Z. mobilis* found that ethanol had no major effects on membrane fatty acid composition but induced changes in phospholipid composition, including a decrease in phosphatidylethanolamine and phosphatidylglycerol and an increase in cardiolipin and phosphatidylcholine content [29]. Another study reported similar changes in phospholipid abundance in response to ethanol, but also observed significant changes in the levels of membrane fatty acids [30].

The physiological effects of isobutanol on *Z. mobilis* remain poorly understood and have yet to be explored. In this study, we utilized a systems-level approach comprising LC–MS/MS-based lipidomics, metabolomics, and shotgun proteomics, to investigate how exposure to ethanol and isobutanol impact the lipid membrane composition and overall physiology of *Z. mobilis*. Our analysis revealed intricate alterations in membrane phospholipid and fatty acid composition resulting from ethanol and isobutanol exposure. Isobutanol exposure also elicited a broad stress response in *Z. mobilis* together with a generalized slowdown of metabolism. This study broadens the current understanding of *Z. mobilis* physiological responses to solvent stress. It will assist in the development of strategies to mitigate the adverse effects of isobutanol solvent and in the systematic engineering of *Z. mobilis* strains with enhanced isobutanol tolerance.

## Results

### Experimental design

*Z. mobilis* (ATCC 31821) was inoculated anaerobically into minimal media at a starting OD_600_ of 0.045. Upon measurable growth, ethanol—at concentrations of 0.3, 0.6, or 0.8 M—or isobutanol—at concentrations of 0.10 or 0.15 M—was added to the cultures. The selected concentrations were determined based on preliminary growth experiments with added ethanol and isobutanol (Additional file 1: Table S1). The cultures were allowed to grow to an OD_600_ of 0.5, at which point samples were collected for lipidomics, proteomics, metabolomics, and microscopy analyses (see Materials and Methods).

### Isobutanol impacts *Z. mobilis* growth and morphology

In the minimal media used in this study, *Z. mobilis* had an average doubling time of 1.7 h. When grown with added ethanol, *Z. mobilis* average doubling times increased to 2.3, 2.9 and 3.3 h for 0.3, 0.6, or 0.8 M added ethanol, respectively (Fig. 1A and Additional file 2: Table S2A). *Z. mobili*s was substantially more susceptible to growth inhibition by isobutanol than ethanol; in the presence of 0.10 and 0.15 M isobutanol, doubling times averaged 2.0 and 5.5 h, respectively (Fig. 1A and Additional file 2: Table S2A).

We used microscopy to evaluate gross morphological alterations when *Z mobilis* was grown with added ethanol or isobutanol (Fig. 1B). We found that increasing concentrations of ethanol did not cause any noticeable changes in cell morphology. However, growth on 0.15 M isobutanol substantially affected cell morphology, causing cells to become more circular and lose their rod-like shape. The average cross-sectional area of *Z. mobilis* cells grown in minimal media was 2.66 µm^2^ and did not change appreciably in the ethanol addition experiments. However, cells grown in the presence of 0.15 M isobutanol increased their average cross-sectional area to 4.25 µm^2^, consistent with the observed changes in morphology.

### Alterations in *Z. mobilis’* membrane lipid composition under ethanol and isobutanol exposure

#### Membrane phospholipid and fatty acid composition under baseline conditions

We used LC–MS/MS-based lipidomics to analyze the fatty acid and phospholipid composition of *Z. mobilis’* lipid membranes when grown on minimal media without added ethanol or isobutanol [31]. LC–MS analysis of saponified *Z. mobilis* membrane phospholipids identified six primary fatty acids: palmitoleic (16:1), myristoleic (14:1), vaccenic (18:1), myristic (14:0), palmitic (16:0) acid, and a putative cyclopropane (19:Cyclo) fatty acid (Table 1 and Additional file 3: Table S3). Among these, vaccenic (18:1) and palmitic (16:0) acids were the most abundant, comprising 79.6% and 11.5% of membrane fatty acids, respectively. Using purified standards, we confirmed the identity of the putative cyclopropane fatty acid as cis-11,12-methyleneoctadecanoic acid (19:cyclo), which constituted 1.6% of membrane fatty acids.

**Table 1:**
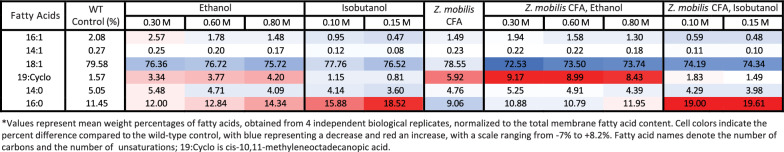
Effects of ethanol, isobutanol, and CFA synthase overexpression on membrane fatty acid composition in *Z. mobilis*

**Fig. 2:**
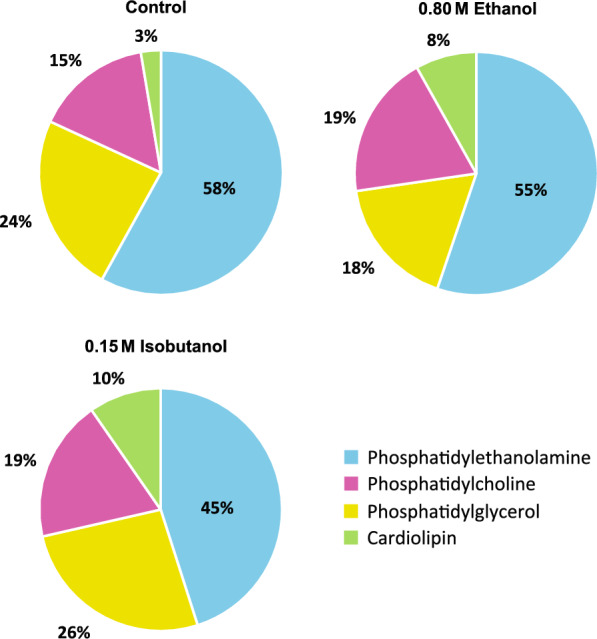
Membrane phospholipid composition of *Z. mobilis* grown anaerobically in minimal media (control), and with added ethanol (0.8 M) or isobutanol (0.15 M). The values displayed represent the percentage of each phospholipid class, which is the sum of all individual phospholipids within that class, relative to the total measured membrane phospholipid content. These values were averaged over four independent biological replicates. The individual phospholipids composition is detailed in Table 2

LC–MS/MS analysis of extracted phospholipids revealed that phosphatidylethanolamine (PE), phosphatidylglycerol (PG), phosphatidylcholine (PC) and cardiolipin (CL) were the predominant lipid classes in *Z. mobilis* (Fig. 2, Table 2 and Additional file 4: Table S4). Phosphatidylethanolamine and phosphatidylglycerol were the most abundant lipid classes, accounting for 58% and 24% of the total measured phospholipids, respectively. In agreement with the observation that vaccenic acid (18:1) is the most abundant fatty acid in *Z. mobilis’* lipid membranes, we found that PE 18:1 18:1 was the most abundant phospholipid, representing 33.8% of all the quantified phospholipids, followed by PG 18:1 18:1 at 16.6% and PG 18:1 18:1 at 8.8% (Table 2 and Additional file 4: Table S4). In combination, these three phospholipids accounted for nearly 60% of all phospholipids. Cyclopropane fatty acids were identified at relatively low abundances in all lipid classes except cardiolipins.

**Table 2:**
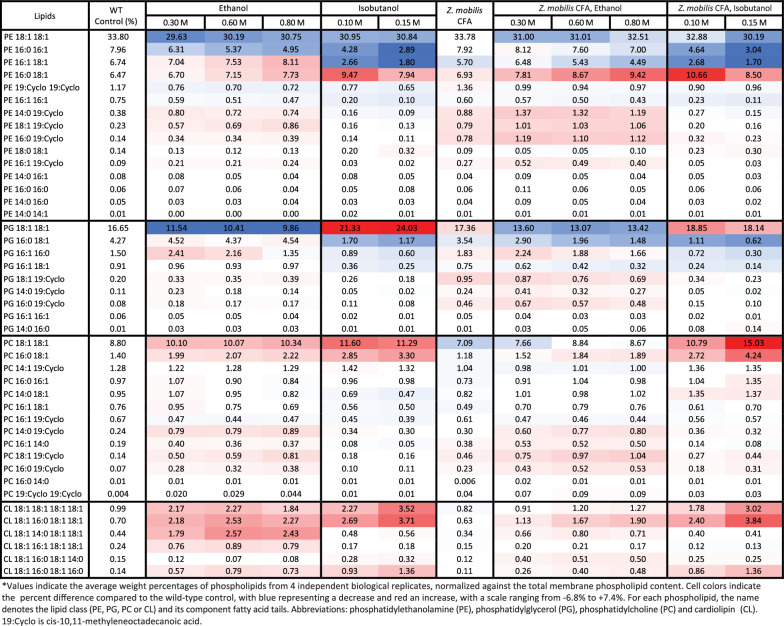
Effects of ethanol, isobutanol, and CFA synthase overexpression on membrane phospholipid composition in *Z. mobilis*

#### Alterations in fatty acid composition under ethanol and isobutanol exposure

We performed saponification of membrane phospholipids to examine changes in membrane fatty acid composition of *Z. mobilis* upon exposure to added ethanol or isobutanol (Table 1 and Additional file 5: Table S5). A notable alteration was the increase in cyclopropane fatty acid (19:Cyclo) content, which rose significantly during ethanol exposure from 1.6% to 4.2% in 0.8 M ethanol. Additionally, ethanol exposure led to an increase in palmitic acid (16:0) from 11.5% to 14.3% in 0.8 M ethanol, although this change did not reach statistical significance. Contrasting with ethanol, growth with added isobutanol resulted in a significant reduction in the levels of cyclopropane fatty acid (19:Cyclo), which fell from 1.6% to 0.8% in 0.15 M isobutanol. Isobutanol exposure also led to a significant increase in the levels of palmitic acid (16:0), which rose from 11.5% to 18.5 in 015 M isobutanol (Table 1).

#### Alterations in phospholipid composition following ethanol and isobutanol exposure

Upon exposure to ethanol or isobutanol, *Z. mobilis* underwent significant and complex alterations in phospholipid abundance (Fig. 2, Table 2 and Additional file 5: Table S5). Exposure to both ethanol and isobutanol resulted in an overall increase in phosphatidylcholine and cardiolipin content. Specifically, cardiolipin content increased from 3% under basal conditions to 8% and 10% in 0.8 M ethanol and 0.15 M isobutanol, respectively. Phosphatidylcholine content increased from 15% under basal conditions to approximately 19% during exposure to both 0.8 M ethanol and 0.15 M isobutanol. The individual phospholipids contributing to the overall increase in phosphatidylcholine and cardiolipin content during ethanol and isobutanol exposure are detailed in Table 2. In contrast to phosphatidylcholine and cardiolipin, overall phosphatidylethanolamine content showed a marked reduction from 58 to 45% during isobutanol exposure. During ethanol exposure, overall phosphatidylethanolamine content also decreased but to a lesser extent than during isobutanol exposure, falling from 58 to 55%. The levels of some phosphatidylethanolamines changed in opposite directions during ethanol and isobutanol addition; for example, PE 16:1 18:1 displayed a large decrease with isobutanol, but it increased slightly with ethanol. Also, ethanol and isobutanol generally had opposite effects on the abundance of phosphatidylglycerols. For instance, while PG 18:1 18:1 decreased during ethanol exposure, it increased with isobutanol. Conversely, PG 16:0 18:1 showed a large decrease with isobutanol but did not change significantly with ethanol.

In agreement with the increase in cyclopropane fatty acid (19:Cyclo) described earlier, we found elevated levels of phosphatidylethanolamines, phosphatidylglycerols, and phosphatidylcholines containing cyclopropane fatty acids during ethanol exposure (Table 2). Conversely, isobutanol exposure was associated with an overall reduction in phospholipids containing cyclopropane fatty acids, consistent with the diminished levels of cyclopropane fatty acid. Interestingly, cyclopropane fatty acids were not identified in cardiolipins during either treatment.

### Proteome remodeling during ethanol and isobutanol exposure

**Fig. 3:**
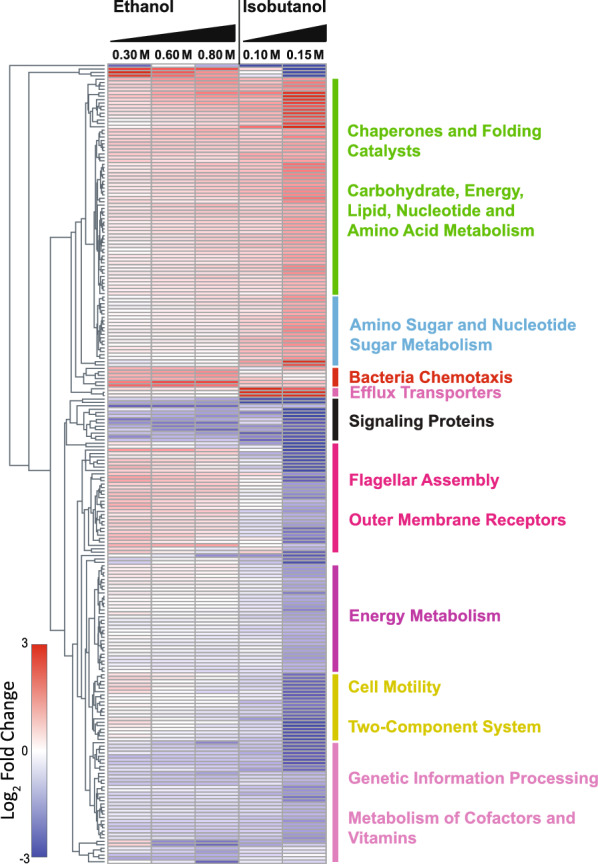
Relative changes in protein abundance upon exposure to ethanol and isobutanol. Only proteins exhibiting a greater than two-fold change (in either direction) with an FDR-adjusted P value of < 0.05 during exposure to either ethanol or isobutanol are shown. The complete proteomics dataset can be found in Additional file 6: Table S6. Each row corresponds to a single protein. Alterations in protein levels are displayed as log2-fold changes relative to unexposed controls, red indicates high relative protein levels and blue indicates low relative protein levels. The data shown represents the average of three independent biological replicates

We performed LC–MS/MS-based proteomic analysis to gain a deeper understanding of the physiological responses elicited in *Z. mobilis* upon exposure to ethanol or isobutanol. Out of the 1890 protein-coding genes in the *Z. mobilis* ZM4 genome [25, 32], relative abundances were determined for 1107 proteins (Additional file 6: Table S6). A total of 222 proteins exhibited significant alterations (FDR-adjusted *P* value < 0.05) and a greater than a two-fold change in abundance in response to isobutanol exposure. During ethanol exposure, 46 proteins showed significant changes with a greater than a two-fold change in abundance (Fig. 3 and Additional file 6: Table S6). Exposure to isobutanol was associated with alterations in various cellular processes, including flagellar assembly, cell motility, chemotaxis, efflux transporters, chaperones, and lipid, nucleotide, and amino acid metabolism. Although exposure to ethanol influenced a similar set of processes, the impact was generally milder.

#### Ethanol and isobutanol induce upregulation of cyclopropane fatty acid synthase

**Fig. 4:**
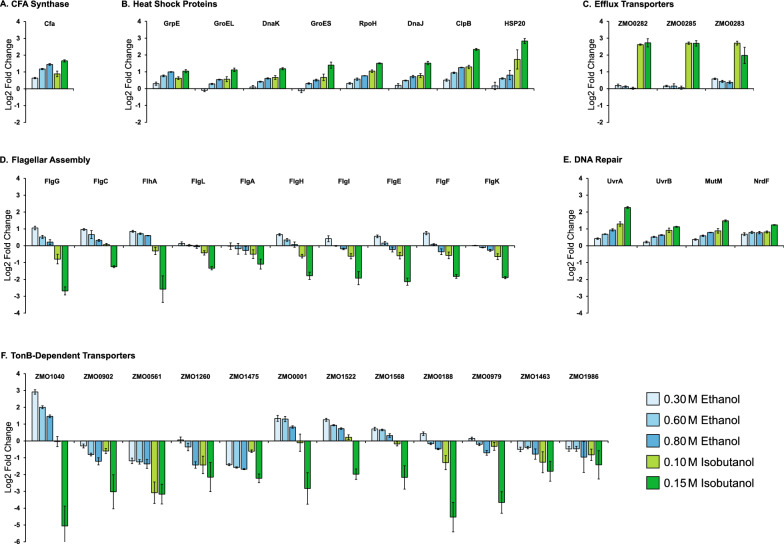
Changes in protein abundance from selected functional categories following ethanol or isobutanol exposure. **A** Cyclopropane Fatty Acid (CFA) Synthase, **B** Heat shock proteins, **C** Efflux transporters, **D** Flagellar assembly, **E** DNA repair, **F** TonB-dependent transporters. Protein names, gene IDs, and the complete proteomics dataset can be found in Table S#. Alterations in protein levels are displayed as log2-fold changes relative to unexposed controls. The data shown represents the average of three independent biological replicates, with error bars representing standard deviation

Cyclopropane-ringed fatty acids (CFAs) serve as key modulators of membrane fluidity and permeability. It has been suggested that the rigid cyclopropane ring in CFAs increases the packing density of the fatty acid chains in the membrane, reducing the availability of free space for the entry of harmful molecules, such as isobutanol [33–36]. We observed a significant increase in the levels of the cyclopropane fatty acid (CFA) synthase protein (ZMO1033) as the concentrations of added ethanol and isobutanol increased (Fig. 4A). When subjected to ethanol, the upregulation of CFA synthase coincided with a rise in the abundance of C19 cyclopropane fatty acids and CFA-containing lipids (Tables 1 and 2). Surprisingly, the increase in C19 cyclopropane fatty acid levels was not detected during isobutanol exposure, even though CFA synthase upregulation was as high as during ethanol exposure (~ 2.7 and ~ 3.2 fold increase for 0.8 M ethanol and 0.15 M isobutanol, respectively). This observation suggests that, while the upregulation of CFA synthase protein levels appears to be a common response to both ethanol and isobutanol exposure, an unknown factor may be inhibiting its activity during isobutanol exposure, thereby impeding the build-up of C19 cyclopropane fatty acids.

#### Isobutanol induces upregulation of heat shock proteins

Heat shock proteins (HSPs) are a class of proteins that are activated in response to various stressors, including high temperature, oxidative stress, or exposure to harmful chemicals [37, 38]. They function primarily as molecular chaperones, aiding in protein folding, averting protein aggregation, and facilitating the repair or disposal of damaged proteins. Upon exposure to isobutanol, there was a significant and concerted elevation in the levels of multiple HSPs, including RpoH (ZMO0749), heat shock protein 20 (ZMO0989), GrpE (ZMO0016), ClpB (ZMO1424), GroES (ZMO1928), DnaK (ZMO0660), DnaJ (ZMO1690), and GroEL (ZMO1929) (Fig. 4B). This substantial rise in HSP levels might be attributed to protein denaturation or aggregation caused by isobutanol. Ethanol exposure also led to a general increase in HSPs; however, the magnitude of this response was smaller compared to isobutanol.

#### Isobutanol induces the upregulation of efflux transporters

Efflux transporters aid in the survival of bacteria in hostile or toxic environments by actively expelling a diverse range of harmful compounds—including toxins, heavy metals, organic solvents, and antimicrobial agents—from within the cell to the external environment [39–42]. We observed a significant elevation in the abundance of efflux transporter proteins in response to isobutanol. Specifically, the levels of efflux transporter proteins ZMO0282, ZMO0283, and ZMO0285 showed an upregulation of over four-fold in the presence of isobutanol (Fig. 4C). However, the presence of ethanol resulted in a small upregulation of only one of these transporter proteins, ZMO0283. The upregulation of efflux transporters suggests that *Z. mobilis* perceives the detrimental effects of isobutanol and activates these transporters to expel the solvent, potentially mitigating cell damage as part of its broader cellular stress response system.

#### Isobutanol and ethanol have distinct effects on flagellar assembly proteins

Flagella are long helical filaments that provide cell motility. They enable bacteria to navigate towards favorable conditions and away from harmful environments [43–45]. We observed a progressive decrease in the levels of flagellar assembly proteins upon exposure to isobutanol. These proteins included FlgG (ZMO0609), FlgC (ZMO0613), FlhA (ZMO0624), FlgL (ZMO0604), FlgA (ZMO0619), FlgH (ZMO0608), FlgI (ZMO0607), FlgE (ZMO0611), FlgF (ZMO0610), and FlgK (ZMO0605) (Fig. 4D). Conversely, exposure to 0.3 M ethanol had an opposite effect, leading to an increase in flagellar assembly protein levels. Notably, this upregulation was less pronounced at ethanol concentrations of 0.6 M and 0.8 M. In fact, for some flagellar assembly proteins, 0.8 M ethanol exhibited no effect or even caused a slight decrease. The observed elevation in flagellar protein levels in response to the lower concentration of 0.3 M ethanol suggests a potential adaptive response, likely aimed at improving motility and facilitating migration towards more favorable conditions. Conversely, the decreased abundance of these proteins upon exposure to isobutanol, or the highest tested ethanol concentration, may represent a survival strategy employed by *Z. mobilis* to optimize energy and resources, redirecting them towards alternative defense mechanisms.

### Ethanol and isobutanol induce the upregulation of the UvrABC repair system

The UvrABC repair system detects and repairs DNA damage. It does this using a complex of UvrA and UvrB subunits to scan the DNA for abnormalities. If damage is detected, the DNA wraps around a UvrB monomer, which uses ATP to help insert a beta-hairpin between the strands, allowing for the detection of lesions [46, 47]. Both ethanol and isobutanol caused an increase in the levels of UvrABC DNA repair system proteins (Fig. 4E). Specifically, UvrA (ZMO1588) and UvrB (ZMO0362) were both upregulated in response to the solvent stress. Concurrently, the DNA repair proteins, MutM (ZMO1187) and NrdF (ZMO0443), were also upregulated in response to the ethanol and isobutanol stress. This upregulation may suggest that both solvents can cause DNA damage that is detrimental to the cell.

#### Isobutanol decreases the levels of TonB signaling proteins

TonB-dependent transporters (TBDTs) are outer membrane proteins that bind and transport ferric chelates called siderophores, as well as other compounds such as vitamin B_12_ and carbohydrates, from the extracellular environment [48–50]. This transport process requires energy in the form of proton motive force [51, 52]. Isobutanol exposure caused a concerted decrease in the levels of TonB-dependent transporter proteins (Fig. 4F). In contrast, ethanol exposure had a complex influence on the expression of Ton B receptor proteins; certain proteins showed increased expression while others showed reduced levels in response to added ethanol. The implications of these alterations in TBDTs expression are currently unclear.

### Exposure to isobutanol elicits widespread metabolic alterations

**Fig. 5:**
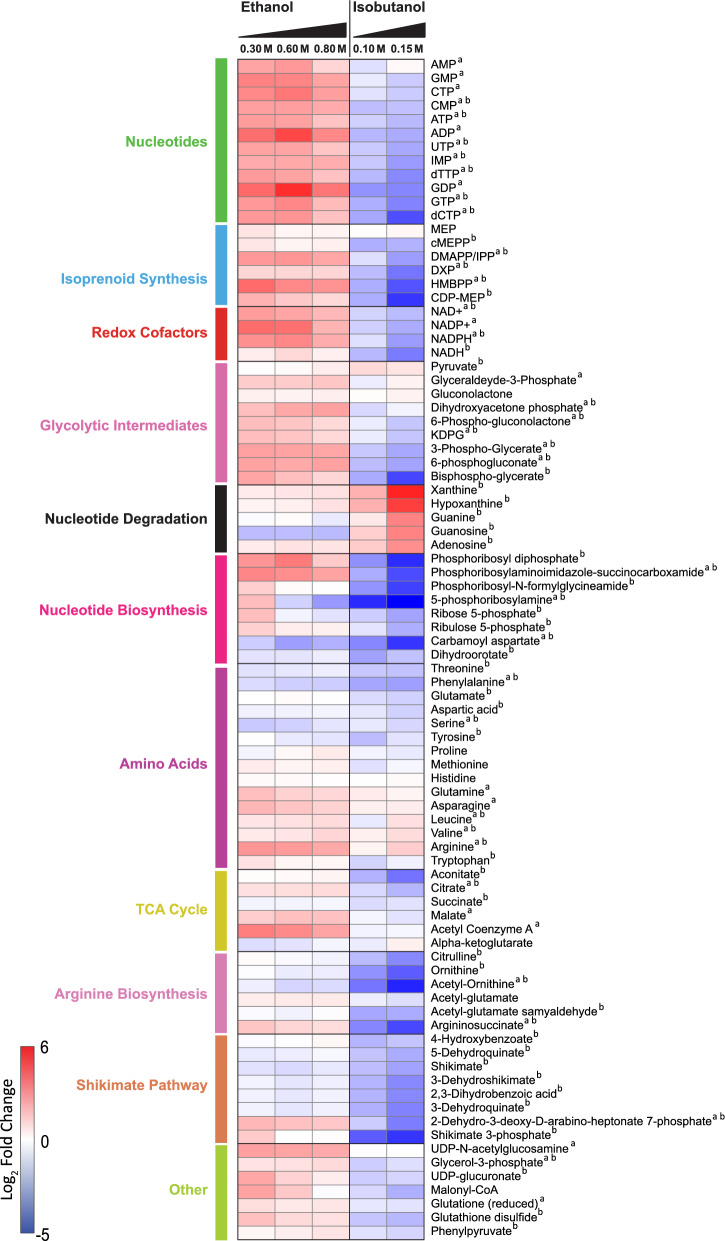
Relative changes in intracellular metabolite levels during ethanol and isobutanol exposure. Only metabolites exhibiting a greater than 1.5 change (in either direction) with a *P* value of < 0.05 during exposure to either ethanol or isobutanol are shown. Each row corresponds to a single metabolite. Alterations in metabolite levels are displayed as log2-fold changes relative to unexposed controls, red indicates high relative metabolite levels and blue indicates low relative metabolite levels. The data shown represents the average of three independent biological replicates. Abbreviations: MEP, 2-C-methyl-d-erythritol 4-phosphate; cMEPP, 2-C-methyl-d-erythritol 2,4-cyclodiphosphate; DMAPP, dimethylallyl diphosphate; IPP, isopentenyl diphosphate; DXP, 1-deoxy-d-xylulose 5-phosphate; HMBPP, 4-hydroxy-3-methylbut-2-enyl diphosphate; CDP-MEP, 4-diphosphocytidyl-2-C-methyl-d-erythritol 2-phosphate; KDPG, 2-dehydro-3-deoxy-d-gluconate 6-phosphate

We used LC–MS metabolomics to measure changes in intracellular metabolite levels during ethanol and isobutanol exposure. This analysis provided relative abundance data for 86 intermediates in primary metabolism distributed across the ED pathway, the tricarboxylic acid (TCA) cycle, the pentose phosphate pathway (PPP), amino acids, and isoprenoid and nucleotide biosynthesis (Fig. 5 and Additional file 7: Table S7). Both ethanol and isobutanol exposure had significant (Fold-change > 1.5 and *P* value < 0.05) widespread effects on intracellular metabolite levels, but these alterations were generally more pronounced with isobutanol. Of the measured metabolites, 60 exhibited significant changes (Fold-change > 1.5 and *P* value < 0.05) in response to 0.15 M isobutanol while 34 metabolites showed significant changes in response to 0.8 M ethanol. Interestingly, for some pathways and metabolite classes, ethanol and isobutanol exposure displayed opposite effects.

#### Isobutanol exposure alters nucleotide metabolism

Exposure to isobutanol resulted in a generalized decrease in nucleotide levels, alongside an increase in nucleotide degradation intermediates. Upon isobutanol exposure, we noted reductions in various nucleotide levels including ATP, GTP, CMP, UTP, and others. We also observed large decreases in nucleotide biosynthesis intermediates during isobutanol exposure, such as phosphoribosyl diphosphate, phosphoribosylaminoimidazole-succinocarboxamide, phosphoribosyl-N-formylglycineamide, 5-phosphoribosylamine, carbamoyl aspartate, Dihydroorotate, and IMP. We also observed depletion of pentose phosphate pathway intermediates, including a threefold decrease in the levels of the pentose sugar ribose 5-phosphate, the pentose component precursor in nucleotides. In parallel to the overall depletion of nucleotides and nucleotide synthesis intermediates, isobutanol exposure resulted in a large increase in the levels of metabolites associated with purine degradation. For instance, the levels of xanthine and hypoxanthine increased over 20-fold, while guanine, guanosine, adenosine increased more than sixfold.

Interestingly, ethanol exposure had an opposite effect to that of isobutanol on nucleotide levels. Instead of depleting nucleotide levels, there was an overall increase in these levels, and it did not prompt a generalized decrease in nucleotide biosynthetic intermediates either. Furthermore, ethanol exposure did not significantly increase the levels of nucleotide degradation products.

#### Isobutanol depletes shikimate pathway, arginine synthesis, and MEP pathway intermediates

During isobutanol exposure, we noted a concerted reduction in the levels of shikimate pathway intermediates, while ethanol did not have this effect. The shikimate pathway provides precursors for aromatic amino acid, folate, and ubiquinone synthesis. All eight of the measured shikimate pathway intermediates were significantly depleted during isobutanol exposure. The aromatic amino acids tyrosine, phenylalanine, and tryptophan also decreased significantly during isobutanol exposure.

The intracellular levels of intermediates in the arginine biosynthesis pathway also diminished during exposure to isobutanol. Specifically, citrulline, ornithine, acetyl-ornithine, and argininosuccinate all decreased more than fivefold. In contrast, during ethanol exposure, only acetyl-ornithine was significantly reduced, albeit to a lesser extent. Interestingly, despite the decrease in biosynthetic precursors, arginine levels increased during isobutanol exposure. Arginine levels were also significantly elevated during ethanol exposure.

Isobutanol exposure also resulted in a generalized decrease in the levels of MEP pathway intermediates. DXP, CDP-MEP, HMBPP, cMEPP, and DMAPP/IPP all exhibited a decrease greater than twofold during isobutanol exposure. In contrast, none of MEP pathway intermediates decreased during ethanol exposure and a few of them showed significant increases instead.

#### Isobutanol depletes ED pathway intermediates and redox cofactors

Notably, Isobutanol exposure also resulted in significantly decreased levels of many intermediates in the ED glycolytic pathway. This included 6-Phospho-gluconolactone, 6-phosphogluconate, KDPG, Bisphospho-glycerate, and 3-Phospho-Glycerate, each of which decreased by more than twofold. Levels of the redox cofactors NADPH, NADH, NAD + , and NADP + all decreased by more than twofold during isobutanol exposure. Neither ED pathway intermediates nor redox cofactors decreased during ethanol exposure; in fact, some of these metabolites experienced a significant increase during exposure to ethanol.

Overall, this metabolomic analysis underscores the extensive impact of isobutanol on intracellular metabolite levels, which is stronger than that of ethanol. The predominant effect of isobutanol exposure is an overall depletion of intermediates across multiple biosynthetic pathways, with only intermediates in nucleotide degradation pathways and some amino acids displaying increased intracellular levels. This suggests that isobutanol has widespread adverse effects on *Z. mobilis*’ primary metabolism, which likely contributes to the observed decline in growth rates in the presence of added isobutanol.

### Isobutanol induces protein aggregation in *Z. mobilis*

**Fig. 6:**
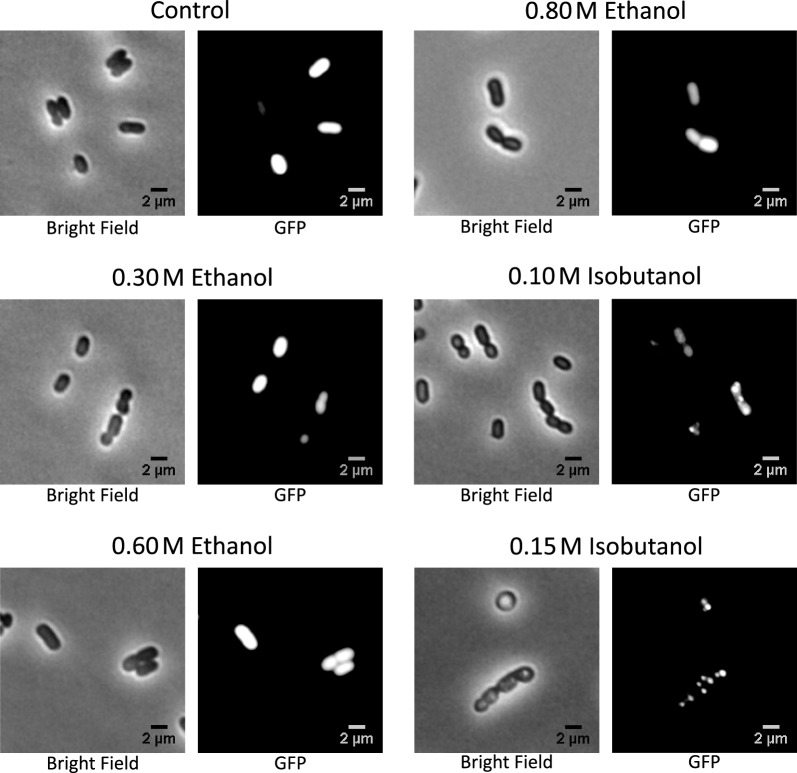
Accumulation of Green Fluorescent Protein (GFP) aggregates in the presence of isobutanol. A *Z. mobilis* strain, engineered to express GFP, was grown in minimal media supplemented with either ethanol (at concentrations of 0.3, 0.6, or 0.8 M) or isobutanol (at concentrations of 0.10 or 0.15 M). Fluorescence microscopy analysis revealed the emergence of GFP aggregates exclusively in the media containing isobutanol

As previously discussed, our proteomic analysis revealed a significant and widespread increase in the levels of heat shock proteins (HSPs) during exposure to isobutanol. We hypothesized that this heightened expression of HSPs might constitute a cellular response to protein denaturation or aggregation caused by isobutanol. To test this, we engineered a *Z. mobilis* strain expressing Green Fluorescent Protein (GFP) and exposed it to both ethanol and isobutanol. Microscopy analysis revealed the formation of GFP aggregates in the presence of isobutanol, which we interpreted as evidence of protein denaturation and aggregation (Fig. 6). This effect was evident with 0.1 M isobutanol and even more pronounced at 0.15 M isobutanol. These findings suggest that isobutanol's toxic effects may be partially mediated through protein denaturation and aggregation, thereby impairing biological functions of enzymes and other proteins. Importantly, no GFP aggregates were observed when ethanol was added, correlating with a much less robust upregulation of HSPs.

### *Z. mobilis* CFA synthase overexpression increases the abundance of cyclopropane fatty acids

To investigate whether the upregulation of cyclopropane fatty acid synthase (CFA synthase) during ethanol exposure contributes to the observed increase in cyclopropane fatty acids in *Z. mobilis* during ethanol exposure, we engineered an IPTG inducible *Z. mobilis* strain to overexpress CFA synthase (see Methods and Materials). Overexpressing *Z. mobilis*’ CFA synthase led to an approximate fourfold increase in 19:Cyclo cyclopropane fatty acid content compared to wildtype baseline levels, accounting for about 6% of the total fatty acids (Table 1). Other fatty acid levels remained relatively stable during CFA synthase overexpression. In the presence of added ethanol (0.3, 0.6 or 0.8 M), the CFA synthase-overexpressing strain showed an average 5.6-fold increase in cyclopropane fatty acid content. Alongside the increased levels of 19:Cyclo cyclopropane fatty acids, CFA synthase overexpression resulted in a significant elevation in PE, PG, and PC lipids containing this fatty acid (Table 2). This effect was more pronounced for certain phospholipids when ethanol was present.

As we showed earlier, isobutanol exposure decreased cyclopropane fatty acid levels. CFA synthase overexpression enabled a restoration of these levels upon isobutanol exposure, but only to levels similar to wildtype baseline (Table 1). During isobutanol exposure, overexpression of *Z. mobilis*‘ CFA synthase led only to a marginal increase in the levels of some cyclopropane fatty acid phospholipids, with several remaining below the control baseline levels (Table 2). Lastly, overexpression of CFA synthase resulted in a minor but significant enhancement in growth rates, averaging ~ 10% increases, in the presence of added ethanol and isobutanol (Additional file 2: Table S2B).

### Overexpression of *C. acetobutylicum *CFA synthase profoundly alters *Z. mobilis* lipid profiles

C. *acetobutylicum* is an anaerobic bacterium that can produce large amounts of butanol during sugar fermentations [53–55]. It was previously reported that this bacterium accumulates cyclopropane fatty acids during butanol production, a process in which CFA synthase is involved [56]. Considering *C. acetobutylicum*'s resilience against butanol, we decided to overexpress the cyclopropane fatty acid synthase from *C. acetobutylicum* in *Z. mobilis.* We reasoned that such an approach could enhance the production of cyclopropane fatty acids in *Z. mobilis* and potentially increase its tolerance to isobutanol.

**Fig. 7:**
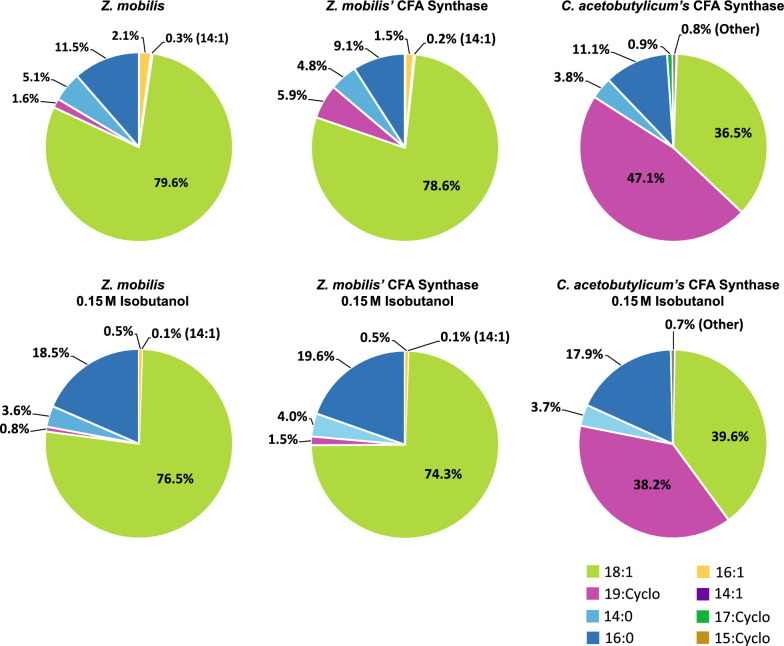
Membrane fatty acid composition of wildtype *Z. mobilis* and strains overexpressing CFA synthase from *Z. mobilis* or *C. acetobutylicum*, grown anaerobically in minimal media (top row) or with 0.15 M added isobutanol (bottom row). The displayed values represent the percentage of each fatty acid in relation to the total measured fatty acid content, averaged over four independent biological replicates. Abbreviations: 16:1, palmitoleic acid; 14:1, myristoleic acid; 18:1, vaccenic acid; 19:Cyclo, cis-11,12-methyleneoctadecanoic acid; 14:0, myristic acid; 16:0, palmitic acid; 17:Cyclo, cis-9,10-methylenehexadecenoic acid; 15:Cyclo, cis-9,10-methylenetetradecenoic acid

**Fig. 8:**
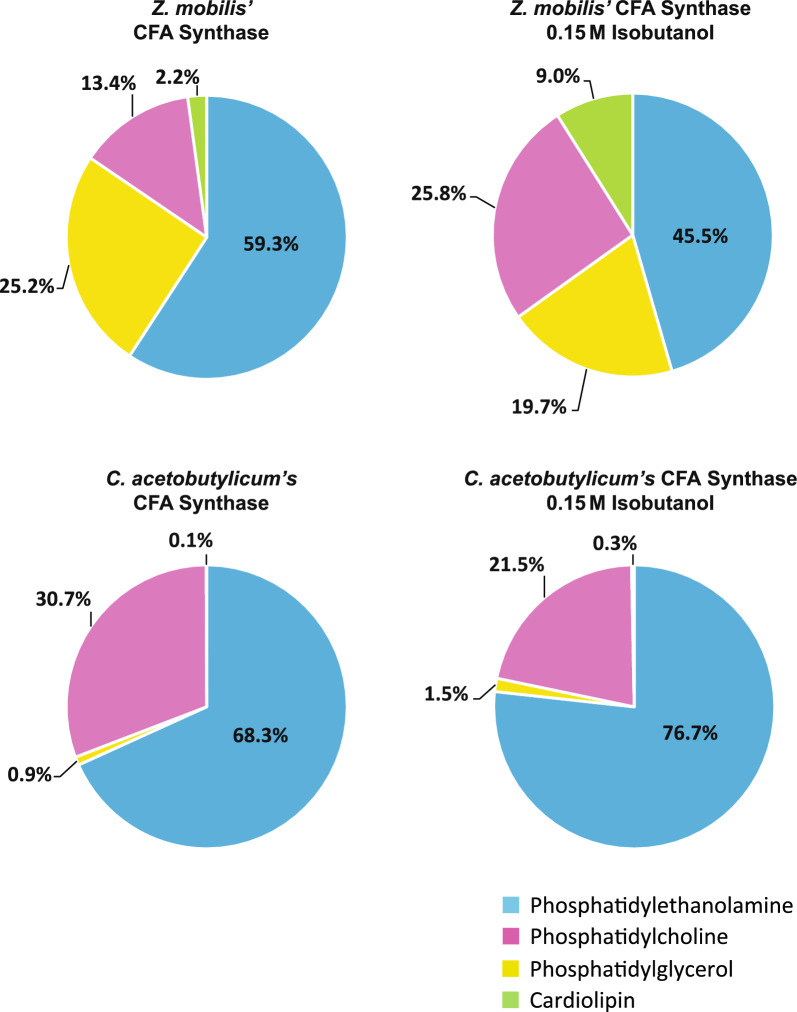
Membrane phospholipid composition of *Z. mobilis* strains overexpressing CFA synthase from *Z. mobilis* or *C. acetobutylicum*, grown anaerobically in minimal media or with 0.15 M isobutanol. The values displayed represent the percentage of each phospholipid class, which is the sum of all individual phospholipids within that class, relative to the total measured membrane phospholipid content. These values were averaged over four independent biological replicates. The individual phospholipids composition is detailed in Table 4

The overexpression of CFA synthase from *C. acetobutylicum* in *Z. mobilis* led to an unexpectedly large increase in the level of C19:cyclo cyclopropane fatty acid, which reached a remarkable 47% of total fatty acids (Fig. 7 and Table 3). This increase was accompanied by a decline in C18:1 content, dropping from 79.6% to 36.5%. Overexpression of CFA synthase from *C. acetobutylicum* also resulted in a substantial increase in phospholipids containing cyclopropane fatty acids (Table 4). Notably, PE 19:Cyclo 19:Cyclo alone accounted for 67% of the total phospholipids, and together with PC 18:1 19:Cyclo and PC 16:0 19:Cyclo, comprised over 95% of measured phospholipids. Surprisingly, unlike PEs and PCs, the levels of PGs containing cyclopropane fatty acids did not increase post-overexpression. The relative abundance of phospholipid classes also changed substantially (Fig. 8). Phosphatidylethanolamine content increased from 58 to 68% and phosphatidylcholine content doubled from 15 to 31%. Conversely, there was a drastic reduction in phosphatidylglycerol and cardiolipin content, which both decreased to less than 1% relative abundance.

**Table 3:**
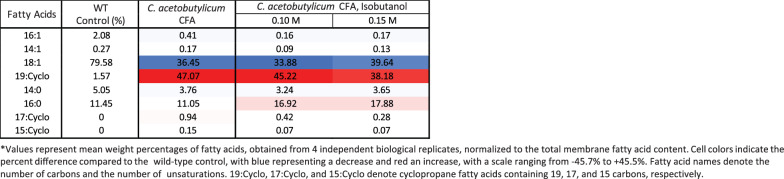
Effects of *C. acetobutylicum*’s CFA synthase overexpression on membrane fatty acid composition in *Z. mobilis*

**Table 4:**
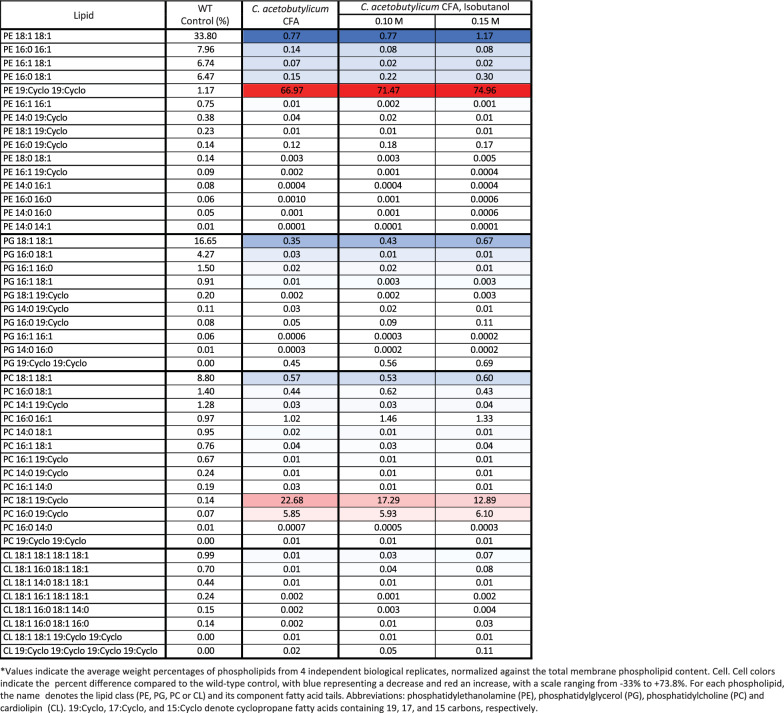
Effects of *C. acetobutylicum*’s CFA synthase overexpression on membrane phospholipid composition in *Z. mobilis*

Exposing the strain overexpressing CFA synthase from *C. acetobutylicum* to 0.15 M isobutanol resulted in only a modest reduction in the C19:cyclo cyclopropane fatty acid content, which still reached about 38%. Similarly, although isobutanol exposure led to a reduction in PC 18:1 19:Cyclo levels, from 22.7% to 13%, the level of PE 19:Cyclo 19:Cyclo actually increased from 67 to 75%. These findings suggest that the CFA synthase from *C. acetobutylicum* is comparatively resistant to isobutanol inhibition, unlike the CFA synthase native to *Z. mobilis*.

Notwithstanding the pronounced increase in cyclopropane fatty acid content due to overexpression of CFA synthase from *C. acetobutylicum*, the engineered strain did not exhibit improved growth rates during isobutanol exposure compared to the strain overexpressing *Z. mobilis*’ CFA synthase (Additional file 2: Table S2C).

## Discussion

### *Z. mobilis* exhibits heightened sensitivity to isobutanol relative to ethanol

*Z. mobilis* exhibited substantially greater susceptibility to inhibition by isobutanol than by ethanol, as evidenced by more pronounced alterations in its proteome, metabolome, and overall cell morphology when exposed to higher concentrations of isobutanol. The heightened toxicity of isobutanol might be ascribed to its larger carbon chain, which confers increased hydrophobicity [57]. This enhanced hydrophobicity may enable isobutanol to penetrate and disrupt lipid membranes more efficiently, leading to cell leakage, and might also enhance its ability to denature proteins crucial for microbial survival and function [58–60]. Moreover, the heightened vulnerability of *Z. mobilis* to isobutanol may arise from its limited exposure to this compound in natural environments, as opposed to ethanol, which commonly reaches high concentrations during fermentation. Consequently, *Z. mobilis* may not have evolved protective mechanisms against the deleterious effects of isobutanol.

### Alterations in lipid membrane composition under ethanol and isobutanol exposure

The membrane phospholipid and fatty acid composition of *Z. mobilis* presented in this study aligns with previous research [29, 61, 62]. Similarly, the observed alterations in phospholipid profiles during ethanol exposure are consistent with findings from earlier studies [29, 30]. However, this study is the first to document a detailed analysis of membrane lipid composition broken down by individual phospholipid species, a task enabled by non-targeted LC–MS/MS analysis [31]. This study also uniquely documents the effects of isobutanol exposure on the lipid membrane composition of *Z. mobilis*.

Exposure to both ethanol and isobutanol significantly altered membrane phospholipid and fatty acid composition. Notably, high ethanol concentrations led to a marked increase in cyclopropane fatty acid content, which correlated with increased levels of cyclopropane fatty acid synthase (CFA synthase). There is limited research regarding the effects of isobutanol on bacterial membrane composition. However, previous studies have reported a significant rise in membrane cyclopropane fatty acid levels in *Clostridium acetobutylicum* when exposed to butanol, a solvent with similar chemical properties to isobutanol [63, 64]. It has been recently proposed that cyclopropane fatty acids enhance the chemical and physical stability of membranes while increasing the tightness of packing within lipid bilayers, which decreases their permeability to potentially harmful compounds such as alcohols [33]. Increased levels of membrane cyclopropane fatty acids have been associated with increased tolerance to osmotic stress, high temperature, acidic pH, and high alcohol concentrations in a number of bacteria [34, 36, 56]. Our findings suggest that the upregulation of cyclopropane fatty acids in *Z. mobilis* represents a regulated defense mechanism against solvent stress. We engineered a *Z. mobilis* to overexpress CFA synthase, which increased cyclopropane fatty acid content in all lipid classes. This change was associated with a modest yet significant improvement in growth rates in the presence of added ethanol or isobutanol.

Interestingly, exposure to isobutanol led to a decrease in cyclopropane fatty acid content despite robust upregulation of CFA synthase to levels similar to those attained during ethanol exposure. The reason behind this contradictory outcome is unclear. One possibility is that the denaturing effects of isobutanol directly impair CFA synthase activity. Alternatively, structural changes to the membrane induced by isobutanol might hinder CFA synthase's action on phospholipid fatty acid chains. Previous research in *E. coli* showed that an unidentified protease, transcribed from an RNA polymerase sigma factor RpoH-dependent promoter, degrades CFA synthase [65]. This prompted us to investigate whether the reduction in cyclopropane fatty acid content in *Z. mobilis* during isobutanol exposure was linked to the concurrent significant rise in RpoH sigma factor levels (Additional file 6: Table S6). However, overexpressing RpoH (ZMO0749) did not significantly alter cyclopropane fatty acid content in the membrane, suggesting that upregulation of this sigma factor does not lead to reduced CFA synthase activity in *Z. mobilis*.

Another notable alteration taking place during exposure to ethanol and isobutanol was an increase in cardiolipin content. These specialized phospholipids, characterized by their unique molecular structure comprising four fatty acid chains, are proposed to play a role in maintaining the structural integrity of the cell membrane under stressful conditions. Specifically, previous studies have shown that the proportion of cardiolipin in bacterial lipid membranes increases during cell swelling or shrinkage associated with osmotic stress [59, 66]. Our microscopy analysis revealed that *Z. mobilis* experiences cell swelling during isobutanol exposure. Therefore, the observed increase in cardiolipin content may represent a regulated response aimed at enhancing the membrane's resistance to rupture and protecting the cell from lysis.

As a Gram-negative alphaproteobacterium, *Z. mobilis* possesses a cell envelope consisting of an inner membrane, a thin peptidoglycan layer, and an outer membrane. The lipid extraction method applied in this study extracts all phospholipids from the cell. Therefore, the reported phospholipids and fatty acids profiles, both under baseline conditions and during ethanol and isobutanol exposure, represent the combined composition of *Z. mobilis*' inner and outer lipid membranes. Future studies could explore whether solvent stress differentially impacts the inner and outer membranes. Additionally, future research could also investigate changes in other membrane components such as hopanoids or lipopolysaccharides, which this study did not address.

### Isobutanol induces a generalized stress response and metabolic slowdown

Our proteomic analysis revealed that isobutanol elicits a broad stress response in *Z. mobilis*, marked by the upregulation of heat shock proteins (HSP), efflux transporters, DNA repair mechanisms, as well as the downregulation of cell motility proteins. The large increase in HSP levels during isobutanol exposure is likely a response to protein denaturation and aggregation. Consistent with this, we found that isobutanol, but not ethanol, causes visible protein aggregation in *Z. mobilis.* This provides further support to the idea that isobutanol toxic effects are partially mediated through protein denaturation and aggregation, impairing the activity of enzymes and other proteins. The accompanying upregulation of efflux transporters indicates that *Z. mobilis* perceives the detrimental effects of isobutanol and activates these transporters to expel the solvent. The upregulation of DNA repairs systems, coupled with large increases to the levels of nucleotide degradation intermediates, suggests that another mechanism of isobutanol toxicity is damage to chromosomal DNA. The decrease in the levels of flagellar assembly proteins upon exposure to isobutanol, may represent an effort to optimize energy and resource allocation, redirecting them toward the previously mentioned repair and defense mechanisms.

Our metabolomic analysis suggested that isobutanol induced a generalized slowdown in metabolic activity, encompassing numerous biosynthetic pathways as well as glucose catabolism. These widespread metabolic alterations might be directly attributed to the protein denaturing effects of isobutanol, but they could also be part of a regulatory response to redirect cellular resources and energy away from biomass production and towards cellular repair and survival processes.

### Overexpression of CFA synthase from *C. acetobutylicum*

Remarkably, the CFA synthase from *C. acetobutylicum* was substantially more effective at increasing membrane cyclopropane fatty acid content compared to *Z. mobilis*’ CFA synthase. The reason for this pronounced effectiveness is currently unknown. A conceivable explanation might be resilience of the heterologous CFA gene/protein from *C. acetobutylicum* against native regulatory mechanisms present in *Z. mobilis*, whether they are transcriptional or post-translational. Alternatively, the CFA synthase from *C. acetobutylicum* might possess intrinsically higher catalytic activity compared to its *Z. mobilis* counterpart. The percentage protein sequence identity shared between the CFA synthases from *Z. mobilis* and *C. acetobutylicum* is only 38.8%, differences in amino acid sequence and structure may underlie the differences in overexpression phenotypes.

The CFA synthase from *C. acetobutylicum* was comparatively more resistant to isobutanol inhibition than its *Z. mobilis* counterpart. Is it possible that CFA synthase from *C. acetobutylicum* has evolved to withstand denaturing effects of butanol, since this solvent is produced at high levels by this bacterium. This tolerance to butanol might translate into tolerance against isobutanol, given the chemical similarity between the two compounds. Further research is required to test these hypotheses.

It is noteworthy that the substantial increase in cyclopropane fatty acid content resulting from the overexpression of CFA synthase from *C. acetobutylicum* did not translate into increased tolerance to isobutanol. This contrast with overexpression of CFA synthase from *Z. mobilis,* in which a much smaller increase in cyclopropane fatty acid content correlated with a slight improvement in growth rates during isobutanol exposure. A potential explanation for this observation might be that such a large increase in cyclopropane fatty acid content is counterproductive to membrane stability and permeability. Alternatively, the depletion of phosphatidylglycerol and cardiolipin in the membrane seen during the overexpression of CFA synthase from *C. acetobutylicum* might counteract the potential benefits of increased cyclopropane fatty acid content.

### Overexpression of other stress proteins

In addition to the overexpression of CFA synthase, we explored whether the overexpression of certain stress response proteins, identified as upregulated during isobutanol exposure, could enhance *Z. mobilis* tolerance to this solvent. We engineered *Z. mobilis* to overexpress the RNA polymerase factor sigma-32 (RpoH, ZMO0749), the heat shock protein Hsp20 (ZMO0989), and a Resistance-Nodulation-Division (RND) efflux pump (ZMO0282, ZMO0283, ZMO0285). While the overexpression of heat shock protein Hsp20 resulted in a modest growth rate improvement of approximately 10% under 0.1 M isobutanol exposure, the overexpression of the other proteins did not lead to increased growth rates during exposures to 0.1 or 0.15 M isobutanol. These findings indicate that effectively enhancing *Z. mobilis* resistance to isobutanol toxicity might require a coordinated upregulation of multiple repair and defense mechanisms, mirroring the changes identified in our proteomic analysis (Fig. 4). Alternatively, mutagenesis and adaptive laboratory evolution could be employed to strengthen *Z. mobilis* resilience against isobutanol.

## Conclusion

In summary, this study provides a comprehensive, systems-level evaluation of the impact of ethanol and isobutanol exposure on the lipid membrane composition and overall physiology of *Z. mobilis*. The generalized stress response and slowdown of metabolism elicited by isobutanol exposure in *Z. mobilis* help explain the decreased growth and fermentation rates under this condition. It is plausible that isobutanol toxicity poses a significant obstacle to its production in engineered isobutanologenic *Z. mobilis* strains. The insights gained from this study will guide engineering of *Z. mobilis* towards the creation of isobutanol-tolerant strains that can better serve as robust platforms for the industrial production of isobutanol from lignocellulosic sugars.

## Materials and methods

### Media composition and growth conditions

*Zymomonas mobilis* ZM4 (ATCC 31821) was grown anaerobically on a rich medium plate for 3 days at 30 °C. A single colony from the plate was utilized to inoculate 6 mL of rich medium. 20 mL ZM4 minimal medium inoculated with 60 µL overnight rich media culture and grown for 16 h. This culture was then used to inoculate three to four 20 mL minimal medium culture per condition to a starting optical density of 0.05 (measured at 600 nm). The cells were grown under different conditions to an O.D_600_ of 0.500 in an anaerobic glove bag before 5 mL being collected and centrifuged for 10 min at 4400 rpm (Allegra X-30R, Beckman Coulter). The pelleted cells were flash-frozen and stored at − 80 °C for lipidomics and proteomics analysis. The concentrations of the stressors were tested to determine resistance (Additional file 1: Table S1). The anaerobic glove bag had an atmosphere of 5% H_2_ and 5% CO_2_, and 90% N_2_; oxygen level was kept < 50 ppm. Rich media plates were prepared using 10 g/L yeast extract, 2 g/L KH_2_PO_4_, 18 g/L agar, and 20 g/L glucose, culminating in a pH of 5.72. Minimal medium contained 1 g/L K_2_HPO_4_, 1 g/L KH_2_PO_4_, 0.5 g/L NaCl, 1 g/L NH_4_SO_4_, 0.2 g/L MgSO_4_ 7H_2_O, 25 mg/L Na_2_MoO_4_ 2H_2_O, 2.5 mg/L FeSO_4_ 7H_2_O, 20 mg/L CaCl_2_ 2H_2_O, 1 mg/L Calcium Pantothenate, and 20 g/L glucose, culminating in a pH of 6.45. For solvent exposure experiments, ethanol was added to the media at 0.3, 0.6 and 0.8 M and isobutanol was added at 0.1 and 0.15 M.

### Lipid extractions

The frozen pellets were thoroughly washed with 500 µL of water and then pelleted within a 2 mL glass vial (Thomas Fisher, 1234R80). Next, 300 µL of a butanol/methanol solution in a 3:1 (v/v) ratio was gently introduced using a glass digital analytical syringe (VWR, 97049-424). Following this, the solution was vigorously vortexed for 30 s and subjected to further mixing with a foam tube holder for 10 min [67]. In a subsequent step, 150 µL of a hexane/ethyl acetate solution in a 3:1 (*v*/*v*) ratio was added, vortexed for 30 s, and mixed for 5 min; this process was repeated twice, resulting in a total of 300 µL of the hexane/ethyl acetate 3:1 (*v*/*v*) solution being added. The reaction was concluded by the addition of 300 µL of a 1% acetic acid solution [67]. After vortexing for 30 s and mixing for 5 min, the solution was centrifuged at 4 °C for 10 min at 4400 rpm. 200 µL of the upper layer was collected for lipid analysis and fatty acid saponification. The collected layer was then dried under a stream of nitrogen gas. The resulting dried lipids were reconstituted in 55 µL of methanol, with 45 µL being used for LC–MS analysis.

### Fatty acid saponification

In a 2 mL glass vial (Thomas Fisher, 1234R80), the dried 200 µL lipid layer was reconstituted by adding 1 mL of a methanol/water solution with a ratio of 9:1 (*v*/*v*) and containing 0.3 M potassium hydroxide [68]. The samples were then subjected to incubation in a water bath at 80 °C for 1 h. Following incubation, 100 µL of formic acid were introduced to acidify the solution. To facilitate phase separation, 900 µL of hexane were added, followed by vortexing for 1 min and subsequent centrifugation at 4400 rpm for 5 min [68]. Subsequently, 600 µL of the uppermost layer were carefully transferred to a new glass vial and dried using nitrogen gas. The resulting sample was then reconstituted with 100 µL of a solvent composed of acetonitrile, isopropanol, and water in a 65:30:5 (*v*/*v*) ratio. For LC–MS analysis, 80 µL of this reconstituted solution were utilized.

### Metabolite extractions

Intracellular metabolites were extracted in the anaerobic chamber by vacuum-filtering 10 mL of cell culture at an O.D_600_ of 0.500 on a hydrophilic nylon filter (Millipore; catalog no. HNWP04700) placed on a sintered glass funnel. To quench metabolic reactions and extract metabolites, the filter was submerged cell face down into a plastic petri dish (5.5-cm diameter) filled with 1.5 mL of cold HPLC grade acetonitrile/methanol/water 40:40:20 (v/v) which was kept on frozen aluminum blocks [69, 70]. This process simultaneously lysed the cells, quenched metabolism, and dissolved intracellular metabolites. The cells were then washed off the filters with the extraction solvent in the petri dish. The solvent was collected in an Eppendorf 1.5 mL tube and centrifuged at 20,000 rcf for 10 min at 4 °C. The supernatant was collected for LC–MS analysis.

### Cloning

CFA synthase from *Z. mobilis* and *C. acetobutylicum* was cloned into the pRL814 plasmid, generously provided by Robert Landick (Professor, UW-Madison, Department of Biochemistry, Great Lakes Bioenergy Research Center). This plasmid originated from a pIND4 fragment [71], housing the lacIq gene and PT7A1-O34, and a pRH52 fragment containing gfp, the pBBR-1 broad host origin of replication, and aadA for spectinomycin resistance [19]. To assemble these constructs, primers were meticulously designed using the NEB builder software (https://nebuilderv1.neb.com/), enabling the amplification of gene and plasmid backbone fragments from template DNA, be it genomic or plasmid DNA. Each gene segment was equipped with a unique ribosome binding site (RBS) ranging from 20 to 30 base pairs in length, determined with the RBS Library Calculator [72–77]. These DNA fragments were designed to overlap by 20–30 base pairs and were seamlessly integrated into the pRL814 plasmid. Employing Gibson assembly [78] reactions with "Hi-Fi" reagents from NEB, these reactions consisted of approximately 0.015 pmol of total plasmid backbone DNA, along with variable gene fragment DNA (ranging from 0.03 pmol to 0.09 pmol), all within a final reaction volume of 25 µl, and they were allowed to run for 1 h at 50 °C. Subsequently, E. coli DH5α cells were transformed, followed by selective screening with 100 μg/mL spectinomycin. The identified colonies underwent scrutiny to confirm successful cloning through plasmid extraction and region-specific gene sequencing. The verified constructs were then introduced into either ZM4 triple (ΔhsdSc, Δmrr, and Δcas3) or quadruple (ΔhsdSc, ΔhsdSp, Δmrr, and Δcas3) mutant strains of ZM4 using a defined conjugation protocol [10, 79, 80]. The resulting ZM4 colonies were meticulously screened, with conjugation being validated through PCR and sequencing. Finally, glycerol stocks of these engineered strains were prepared and stored at − 80 °C.

### LC–MS metabolomics

The general LC–MS method was performed using a Vanquish ultra-high-performance liquid chromatography (UHPLC) system (Thermo Scientific) coupled to a hybrid quadrupole Orbitrap mass spectrometer (Q Exactive; Thermo Scientific). The chromatography was done using a reverse-phase C18 column (Acquity UPLC BEH) (1.7-µm particle size, 2.1-by-100-mm column). Solvent A was 97% H2O and 3% methanol with 10 mM tributylamine (TBA) and ∼10 mM acetic acid for a pH of 8.2. Solvent B was 100% methanol [25]. The total run time was 25 min. Flow rate was held constant at 0.2 mL/min. The chromatography gradient was as follows: 5% solvent B for 2.5 min, linear increase to 95% B over 14.5 min, maintenance at 95% B for 2.5 min, linear decrease back to 5% B over 0.5 min, maintenance at 5% B for 5 min. For the general method, eluent from the column was analyzed by MS from the start of the run until 19 min, at which time the flow was directed to waste for the remainder of the run [81].

### LC–MS/MS Lipidomics

The LC–MS lipid method was performed using a Vanquish ultra-high-performance liquid chromatography (UHPLC) system (Thermo Scientific) coupled to a hybrid quadrupole Orbitrap mass spectrometer (Q Exactive; Thermo Scientific). The chromatography was done using a reverse-phase C18 column (Acquity UPLC CSH) (1.7-µm particle size, 2.1-by-100-mm column). Solvent A was 70% acetonitrile and 30% H2O with 10 mM ammonium acetate and 250 µL acetic acid per liter. Solvent B was 90% isopropanol and 10% acetonitrile with 10 mM ammonium acetate and 250 µL acetic acid per liter. The total run time was 33 min. Flow rate was held constant at 0.4 mL/min. The chromatography gradient was as follows: 2% solvent B for 2 min, linear increase to 30% B over 3 min, linear increase to 85% B for 14 min, linear increase to 99% for 1 min, maintenance at 99% B for 7 min, linear decrease back to 2% B over 1 min, maintenance at 2% B for 5 min. For the general method, eluent from the column was analyzed by MS from the start of the run until 28 min, at which time the flow was directed to waste for the remainder of the run. The quantitation of phospholipids was conducted via external calibration using the following standards: cardiolipin 16:0 16:0 16:0 16:0, cardiolipin 18:1 18:1 18:1 18:1, phosphatidylethanolamine 14:0 14:0, phosphatidylethanolamine 16:0 16:0, phosphatidylethanolamine 18:0 18:1, phosphatidylethanolamine 16:0 18:1, phosphatidylethanolamine 18:1 18:1, phosphatidylglycerol 14:0 14:0, phosphatidylglycerol 16:0 16:0, phosphatidylglycerol 18:0 18:0, phosphatidylglycerol 18:1 18:1, phosphatidylcholine 16:0 18:1, and phosphatidylcholine 18:1 18:1. These lipid standards were purchased from Avanti Polar Lipids, Inc. For many of the identified phospholipid species, purified standards are not commercially available. In such cases, the most similar standard, in terms of phospholipid class, chain length, and structure, was used for quantification purposes.

### LC–MS analysis of fatty acids

The fatty acid LC–MS method was performed using a Vanquish ultra-high-performance liquid chromatography (UHPLC) system (Thermo Scientific) coupled to a hybrid quadrupole Orbitrap mass spectrometer (Q Exactive; Thermo Scientific). The chromatography was done using a reverse-phase C18 column (Acquity UPLC CSH) (1.7-µm particle size, 2.1-by-100-mm column). Solvent A was 60% acetonitrile and 40% H2O with 10 mM ammonium acetate and 250 µL acetic acid per liter. Solvent B was 90% isopropanol and 10% acetonitrile with 10 mM ammonium acetate and 250 µL acetic acid per liter. The total run time was 33 min. Flow rate was held constant at 0.4 mL/min. The chromatography gradient was as follows: 2% solvent B for 2 min, linear increase to 30% B over 16 min, linear increase to 85% B for 5 min, linear increase to 99% for 1 min, maintenance at 99% B for 3 min, linear decrease back to 2% B over 1 min, maintenance at 2% B for 5 min. For the general method, eluent from the column was analyzed by MS from the start of the run until 28 min, at which time the flow was directed to waste for the remainder of the run. The quantitation of fatty acids was conducted via external calibration using the following standards: 16:1, palmitoleic acid; 14:1, myristoleic acid; 18:1, vaccenic acid; 19:Cyclo, cis-11,12-methyleneoctadecanoic acid; 14:0, myristic acid; and 16:0, palmitic acid. Fatty acid standards were purchased from Avanti Polar Lipids, Inc and Matreya, LLC. For some of the fatty acids identified, purified standards are not commercially available. In such cases, the most similar standard, in terms of chain length and structure, was used for quantification purposes.

### LC–MS/MS lipidomics data processing

Raw file data sets were processed using MZmine 2 v2.37 [82]. The mass detection was used with a retention time of 0.20–26 min. The noise level was 1.0E4 for mass detector in centroid. The chromatogram builder was used with a retention time of 0.20–26.00, minimum time span of 0.05 min, a minimum of height of 5.0E4 and m/z tolerance of 0.005 m/z and 10.0 ppm. The chromatogram deconvolution was conducted with the local minimum search algorithm. The chromatographic threshold was 0.02%; the search minimum in retention time (RT) range was 0.05 min, a minimum relative height of 0.02% and a minimum ratio of peak top/edge of 3. Peak duration range 0.05–1.50 min. Isotopologues were grouped using the isotopic peaks grouper algorithm with an m/z tolerance of 0.005 m/z and 10.0 ppm, an RT tolerance of 0.05 absolute min and maximum charge of 2. The representation isotope was set to most intense. A peak alignment step was performed using the join aligner module (m/z tolerance = 0.005 m/z and 10.0 ppm, weight for m/z = 20, RT tolerance = 0.1 absolute min, weight for RT = 20). A gap filling module was used with the same RT and m/z range gap filling (m/z tolerance = 0.005 m/z and 10.0 ppm). The resulting peak list was then filtered using the peak list row filter module with a minimum peak in a row of 0.75 and a minimum peak in an isotope pattern of 2. The peak list was then exported to *.csv using the module “Export to CSV file” with the export common elements (Export row ID, Export row m/z, Export row retention time, Export row identity (all IDs), Export row comment, Export row number of detected peaks) and export data file elements (Peak Status, Peak m/z, Peak RT, Peak Height, Peak area, Peak charge, Peak FWHM). The peak list was annotated using LipiDex [31].

Data analysis was conducted with the El-MAVEN software (Elucidata) [83], leveraging retention times matched against authenticated pure standards for compound identification. Fold changes in lipid, fatty acid or metabolite concentrations under various growth conditions were quantified relative to *Z. mobilis* cultured in minimal media. Signal intensities were subjected to a log2 transformation, followed by two-tailed t-tests with equal variance assumptions to calculate corresponding P values.

### Protein extraction

During protein extraction, 10 mL of bacterial culture were collected and centrifuged for 2.5 min at 4000 × g at 4 °C. The supernatant was discarded, and the cell pellets were frozen in liquid nitrogen and stored at − 80 °C until further analysis. For proteomics analysis, the cell pellets were thawed and lysed by resuspending in 6 M guanidine hydrochloride [25, 81, 84]. The samples went through three cycles of heating to 100 °C for 5 min and re-equilibration to room temperature for 5 min. Determination of total protein concentration was carried out using a Pierce bicinchoninic acid (BCA) protein assay kit (Thermo Scientific), and 50–100 µg of protein was utilized for further processing. Methanol was introduced to achieve a final concentration of 90%, followed by centrifugation at 15,000 × g for 5 min. Subsequently, the supernatant was removed, and the protein pellets were desiccated for 10 min. These pellets were then reconstituted in 200 µL of lysis buffer [comprising 8 M urea, 100 mM Tris (pH 8.0), 10 mM tris(2-carboxyethyl)phosphine (TCEP) hydrochloride, and 40 mM chloroacetamide] to induce denaturation, reduction, and alkylation of proteins. The resuspended proteins were diluted to 1.5 M urea in 100 mM Tris (pH 8.0). Trypsin was added at a ratio of 50:1 sample protein concentration to trypsin and incubated overnight (approximately 12 h) at room temperature. The trypsinization reaction was terminated by adding 10% trifluoroacetic acid (TFA). Subsequent to protein digestion, each sample underwent desalting using a Strata-X 33 µM polymeric reversed-phase styrene divinylbenzene solid-phase extraction cartridge and was subsequently dried. Prior to LC–MS/MS analysis, the samples were reconstituted in a 0.2% formic acid solution, and peptide concentrations were quantified employing a Pierce quantitative colorimetric peptide assay kit (Thermo Scientific).

### LC–MS/MS proteomics

For every analysis, 2 μg of peptides were loaded onto a 75-μm-inside-diameter (i.d.), 30-cm-long capillary featuring an embedded electrospray emitter and were packed into a C18 BEH column with 1.7-μm particle size. The mobile phases employed were as follows: phase A, containing 0.2% formic acid, and phase B, composed of 0.2% formic acid–70% acetonitrile. Peptides were eluted using a gradient that transitioned from 0 to 75% B over 42 min, followed by a 4 min wash with 100% B and 10 min of equilibration in 100% A, resulting in a comprehensive 60 min gradient. The eluting peptides underwent analysis using an Orbitrap Fusion Lumos mass spectrometer (Thermo Scientific). Survey scans were conducted at a resolution of 240,000, with isolation analysis within the range of 300–1350 m/z and an AGC target of 1e6. Data dependent top-speed (1-s) tandem MS/MS sampling of peptide precursors was activated, with dynamic exclusion set at 10 s for precursors with charge states ranging from 2 to 4. MS/MS sampling was performed by utilizing 0.7-Da quadrupole isolation and fragmentation via higher-energy collisional dissociation (HCD) with a collisional energy value of 25%. The mass analysis was carried out within the ion trap using the "turbo" scan speed, spanning a mass range of 200 to 1200 m/z. The maximum injection time was configured at 11 ms, and the AGC target was set at 20,000.

### Proteomics data analysis

The MaxQuant software (version 1.5.8.3) [85] was employed for the analysis of the raw LC–MS files. The spectra were subjected to a search against a target decoy database using the Andromeda search engine. Label-free quantitation and match between runs were enabled. The MS/MS tolerance was set at 0.4 Da, and all other analysis parameters utilized their default settings. The peptides were organized into protein groups and filtered to meet a 1% false discovery rate (FDR) criteria based on the target decoy method. The log2-transformed label-free quantitation intensities were further manipulated to calculate log2 fold change values, comparing them either to Z. mobilis overexpressing GFP or to the background signal established from randomly generated signals falling within the noise range.

### Microscopy

GFP overexpressing *Z. mobilis* was grown at a temperature of 30 °C under multiple conditions. These conditions included minimal media with ethanol concentrations of 0.3, 0.6, and 0.8 M, as well as isobutanol concentrations of 0.1 and 0.15 M. When the culture reached an optical density (O.D. 600) of 0.500, a 1 µL aliquot was directly applied to a 1% agar-minimal media-coated slide. The slide was left to air-dry for 5 min. These samples were promptly subjected to imaging utilizing the Olympus IX-83 inverted microscope (manufactured by Olympus), equipped with a 60X phase-contrast objective and fluorescence filters (excitation at 470/20 nm, a dichroic mirror at 485 nm, and emission at 515/50 nm for GFP).

### Supplementary Information

**Additional file 1: Table S1.** *Z. mobilis'* final growth at varying isobutanol concentrations.

**Additional file 2: Table S2.** Doubling times of *Z. mobilis*' CFA Synthase overexpressing strain grown in varying ethanol and isobutanol concentrations.

**Additional file 3: Table S3.** Fatty acid composition of *Z. mobilis* (basal conditions)

**Additional file 4: Table S4.** Lipid composition of *Z. mobilis *(basal conditions)

**Additional file 5: Table S5.** P-values of the effects of ethanol, isobutanol, and CFA synthase overexpression on membrane fatty acid composition in *Z. mobilis*. Also shown are the standard deviations corresponding to the fatty acid composition values in Tables 1 and 3.

**Additional file 6: Table S6.** P-values of the effects of ethanol and isobutanol on the proteome in *Z. mobilis*.

**Additional file 7: Table S7.** Effects of ethanol and isobutanol on the metabolome in *Z. mobilis*.

## Data Availability

All data will be made available as Supplementary Information. Bacterial strains will be made available upon request.

## Abbreviations

PE: Phosphatidylethanolamine
PG: Phosphatidylglycerol
PC: Phosphatidylcholine
CL: Cardiolipin
16:1: Palmitoleic acid
14:1: Myristoleic acid
18:1: Vaccenic acid
19:Cyclo: Cis-11,12-methyleneoctadecanoic acid
14:0: Myristic acid
16:0: Palmitic acid
17:Cyclo: Cis-9,10-methylenehexadecenoic acid
15:Cyclo: Cis-9,10-methylenetetradecenoic acid
MEP: 2-C-methyl-d-erythritol 4-phosphate
cMEPP: 2-C-methyl-d-erythritol 2,4-cyclodiphosphate
DMAPP: Dimethylallyl diphosphate
IPP: Isopentenyl diphosphate
DXP: 1-Deoxy-d-xylulose 5-phosphate
HMBPP: 4-Hydroxy-3-methylbut-2-enyl diphosphate
CDP-MEP: 4-Diphosphocytidyl-2-C-methyl-d-erythritol 2-phosphate
KDPG: 2-Dehydro-3-deoxy-d-gluconate 6-phosphate
GFP: Green fluorescent protein
CFA: Cyclopropane fatty acid synthase
